# A Probabilistic Approach for Breast Boundary Extraction in Mammograms

**DOI:** 10.1155/2013/408595

**Published:** 2013-11-10

**Authors:** Hamed Habibi Aghdam, Domenec Puig, Agusti Solanas

**Affiliations:** Department of Computer Engineering and Mathematics, Rovira i Virgili University, 43007 Tarragona, Spain

## Abstract

The extraction of the breast boundary is crucial to perform further analysis of mammogram. Methods to extract the breast boundary can be classified into two categories: methods based on image processing techniques and those based on models. The former use image transformation techniques such as thresholding, morphological operations, and region growing. In the second category, the boundary is extracted using more advanced techniques, such as the active contour model. The problem with thresholding methods is that it is a hard to automatically find the optimal threshold value by using histogram information. On the other hand, active contour models require defining a starting point close to the actual boundary to be able to successfully extract the boundary. In this paper, we propose a probabilistic approach to address the aforementioned problems. In our approach we use local binary patterns to describe the texture around each pixel. In addition, the smoothness of the boundary is handled by using a new probability model. Experimental results show that the proposed method reaches 38% and 50% improvement with respect to the results obtained by the active contour model and threshold-based methods respectively, and it increases the stability of the boundary extraction process up to 86%.

## 1. Introduction

Breasts are soft parts of the body which are normally composed of fatty tissues as well as specialized tissues that produce milk. Breast cancer is a very serious disease. The early detection of the disease increases the success of treatment. However, its early detection is difficult since there are no symptoms during the first stages of breast cancer development. Fortunately, X-ray mammography can reveal small changes in breast tissue [1]. Mammograms can be used to detect characteristic masses and microcalcifications.

We distinguish two kinds of mammographies, namely, *craniocaudal (CC)* where the breast is compressed horizontally and an X-ray image is taken in the direction from head to toe and *mediolateral oblique (MLO)* where the breast is vertically compressed and an X-ray image is taken from the side. Although our method is suitable for both approaches, in this paper, we only concentrate on MLO mammographies since the database (mini-MIAS) that is used in this study only provides this type of mammograms.

To take mammograms, radiographers help patients to position their breast between two small plates where X-rays pass through the tissues of the breast. The plates then compress the breast for a moment to take an X-ray image. Each breast is compressed to a thickness of approximately 6 cm, and an X-ray image is taken perpendicular to the plane of compression [1]. The diverse densities of the breast tissues attenuate the X-rays differently and translate into different degrees of brightness in the final image.

The breast is connected to the pectoral muscle, fatty tissues are located below the skin, and lobules and ducts are located at the center of the breast. There is a phenomenon, known as *ductal carcinoma in situ (DCIS)*, that affects the cells lining the breast ducts. The breast cancer cells are only inside the ducts and lead to the generation of dense areas at the center of the breast (in some women, DCIS may spread into the surrounding breast tissues after some years to become an invasive ductal breast cancer [2]).

As we stated before, during a mammography process, X-rays enter from one side of the breast and exit from the other side. Inside the breast, each tissue attenuates the X-rays to some degree. As a result, the final X-rays attenuation is affected by each tissue inside the breast. The boundary of the compressed breast has a lower thickness in comparison to its inner parts, but its texture remains as the tissue attenuates the X-rays to a lower degree and produces dark areas in the image. In the same way, whenever X-rays pass through tissues in the breast, they are attenuated according to the density of the tissues and produce bright areas in the image.

Due to the attenuation produced by the different densities of the breast tissues, the final image of the breast is characterized by a specific pattern of gray levels in its different regions. In other words, different regions of the mammogram have different textures (note that, since no imaging technique is perfect, we can expect noise to appear on mammograms).

Analyzing mammograms is one of the hardest tasks even for human experts. Hence, there is a considerable need for *computer*-*aided diagnosis* (CAD) systems to help radiologists to detect and diagnose new cases. A complete breast image analysis system must be able to extract boundaries of breast and pectoral muscle and segment them into different parts such as fatty region, dense region, pectoral muscle region, and tape background.

In this paper we propose a probabilistic learning method for tracing the boundaries of the breast and the pectoral muscle. Our method overcomes the problems of thresholding and deformable techniques and provides accurate, applicable, and stable results. In addition, our method does not require preprocessing techniques to remove artifacts or to align the breast image. In contrast with the previously proposed techniques, our method learns the shape information of the mammogram from training mammograms, and, hence, there is no need for a manual determination of parameters. Also, instead of using pixel intensities or edge information, our proposed method utilizes the texture information of each pixel. Experimental results have been obtained by using the mini-MIAS database, and they show that our method is able to extract accurate boundaries even for noisy mammograms.

The rest of the paper is organized as follows. First, we review the current state of the art related to the problem of determining the breast boundary in Section 2. In Section 3, we describe our formulation of breast boundary extraction in a probabilistic framework, and we show how to find the texture, the smoothness, and the prior probability model. This section is completed with a description of the initialization and tracing algorithms. The evaluation criteria and the experimental results are detailed in Section 4. Finally, the paper concludes in Section 5 by pointing out some final remarks and future research lines.

## 2. Previous Works

Generally, there are two different approaches to cope with the extraction of the breast boundary. The first approach is based on the combination of image processing techniques such as thresholding, watershed transformation, morphological operations, and flood fill. The second approach is founded on well-known deformable techniques such as the active contour model and level-set methods.

### 2.1. Image Processing Techniques

 In early studies by Karssemeijer [3], the segmentation of the breast region was done by using thresholding and morphological operations. It was observed that threshold values could be computed using interactive methods in which the users defined threshold values and evaluated the result. This procedure was repeated until the user found an accurate output [4].

In addition, Dehghani and Dezfooli [5] utilized a simple thresholding method to extract the breast region. Similarly, Nagi et al. [6] thresholded the image using a fixed threshold value and then applied a combination of morphological operations to separate the breast region from the background. However, to find the threshold value automatically, Wang and Qin [7] discarded the lowest and highest bins of the histogram and then smoothed it using a low-pass filter. Next, the threshold value was selected as the first left valley left of the peak. Finally, they smoothed the extracted boundary using morphological closing and opening operations. As another threshold-based method, Raba et al. [8] computed *N* different threshold values and found values related to the largest and smallest regions. Then, the final threshold value was computed by evaluating these regions statistically.

Since a mammogram usually contains noise and other artefacts such as labels, it is reasonable to add preliminary steps to remove them from the image. To this end, Tzikopoulos et al. [9, 10], first, aligned the image using the chest wall location. In order to determine the chest wall location, they used the decreasing pixel intensity of the breast tissue. In the second stage of their procedure, noise and artefacts of the image were removed by finding high-intensity pixels of the image and replacing them by black pixels. Also, speckle noise in the image was removed by using median filters. Finally, to find the breast boundary, they thresholded the image using several values and applied the method proposed by Masek [11] to extract the breast boundary.

In another approach, Maitra et al. [12] used the same method proposed by [11] to find the orientation of the image and to remove its noise. Then, they proposed a segmentation method based on pixel intensity called *binary homogeneity enhancement algorithm* to segment the image into 16 gray levels. Kuş and Karagöz [13] also proposed a similar segmentation approach based on texture filters.

Mello and Tenorio [14] addressed the breast boundary extraction problem in a multistage procedure. First, they used a reference image to modify the histograms of the test images such that they obtained histograms more similar to that of the reference image. Then, each test image was downsized to 30 pixels height and resized back to its original size to remove small elements of the image. Next, the contrast of the image was increased, and the absolute difference between it and the histogram-modified image was calculated. After applying another contrast adjustment algorithm, a flood-fill technique filled dark holes of the obtained image. Finally, the breast boundary was extracted by thresholding the image and by applying morphological closing and skeleton methods.

Maysam Shahedi et al. [15] processed the image using a nonlinear diffusion filter and a median filter in order to reduce its noise and then found the proper threshold value iteratively by evaluating the performance of each threshold using the compactness of the growing region. Following this idea, Zhang et al. [16] developed a smart region growing method to solve this problem. To initialize the algorithm, they developed an automatic method to find one seed inside the breast region and another seed outside the breast region. Then, a smart region growing algorithm was applied on the seed inside the breast region, and a noisy breast boundary was obtained. Finally, to smooth the obtained breast boundary, a low-pass fast Fourier transform was applied. In addition, Saha et al. [17] proposed a region growing method based on scale-based fuzzy connectivity. This approach was not fully automatic since some parameters had to be determined empirically.

There are other similar approaches [18–24] which use nonlinear filtering and connected component labeling to remove the noise and artefacts of the image and use threshold values or region growing in conjunction with morphological operation to extract the boundary of the breast. Regardless of their algorithmic similarity, the most common characteristic of the above methods is that they use raw pixel values or the pixel intensities means in small windows so as to extract the boundary of the breast.

### 2.2. Deformable Models

Another popular approach to determining the breast boundary is to use deformable models such as the active contour model or level-set methods. Since the boundary of the breast is a well-defined curve and the background region is more likely to be composed by low-intensity and low-gradient pixels, it is reasonable to use active contour models (snakes) to look for local minima. The basic idea behind active contour models is to find a contour such that the internal and external energy of the contour is minimized. Basically, external energy is calculated as the negative of the gradient of the Gaussian smoothed image.

Similarly, level-set methods start with an initial contour and change it using a simple equation. The amount of change at each point on the contour is determined by using a potential function. Generally, the potential function is zero wherever the gradient of the image is maximum. Ferrari et al. [25] initialized the active contour model using a global thresholding method and then evolved the active contour model (snake) along its normal until it reaches the minimum of its energy function. Similarly, Wirth and Stapinski [26] initialized the algorithm by fitting a piecewise quadratic curve on a dual thresholded image and picked the points at specific interval along this curve.

Differently from traditional models, Thiruvenkadam et al. [27] divided the image into rectangular regions and defined two Gaussian models: one for the pixels of the object and another for the background pixels. Then, instead of searching the location of contour points of the active contour model, they tried to find optimal values for the Gaussian distribution of each rectangular region. In another attempt to obtain accurate results, Yu et al. [28] used a gradient vector flow snake [29] to find the boundary of the breast.

One of the most recent approaches for finding the boundary of the breast is that proposed by Marti et al. [30]. Their method was based on growing the boundary from an initial seed point. The concept was similar to that of a snake except that, instead of global optimization of all boundary points, their method found a single point at each iteration. The similarity between their method and snakes resides in their decision function. It is like an energy function of snakes without integral operators.

To the best of our knowledge, there are only a few studies on the pectoral muscle boundary extraction problem. Mustra et al. [31] used bit depth reduction and wavelet decomposition for finding the pectoral muscle, and Nagi et al. [6] utilized a seeded region growing algorithm to extract the pectoral muscle region. Amongst different approaches, the work done by Kwok et al. [32] is the most significant study in this area. It first defined regions of interest and, then, selected a threshold value in an iterative algorithm. Then, pixels of the pectoral muscle boundary were traced, and a gradient test was performed on them. Finally, a straight line was fitted on the selected pixels, and, then, it was refined with an algorithm known as cliff detection.

## 3. Proposed Method

In a mammogram, the number of dark pixels is generally greater than the number of bright pixels, and, usually, bright pixels are distributed uniformly. This results in a histogram with significant peaks at dark pixels and a near-uniform shape at bright pixels. Due to this fact, finding a proper threshold is a difficult task, and it gets even more difficult in the presence of noise. Clearly, the determination of an inappropriate threshold value would lead to the incorrect selection of larger or smaller areas for the breast.

**Figure 1:**
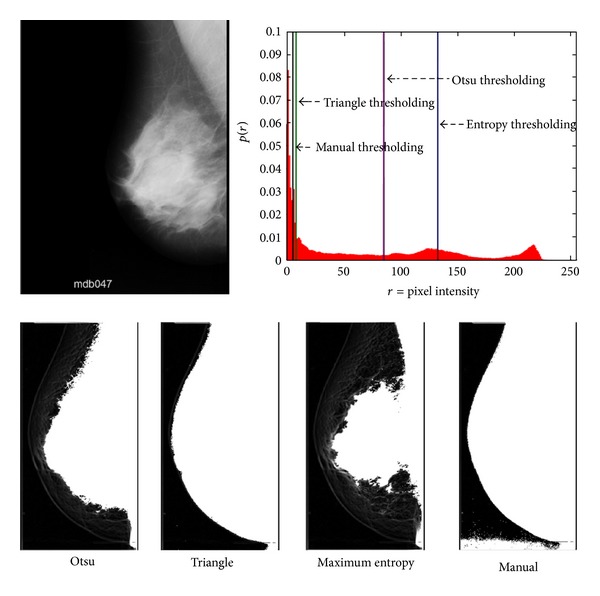
Example of breast extraction using different thresholding methods.

 Threshold-based methods, which try to find the threshold value by optimizing some evaluation function directly on the histogram of the image, can easily fail. This problem is illustrated in Figure 1, in which a mammogram without extra artifacts is shown. It can be observed that, in addition to manual thresholding, we also applied three different automatic thresholding methods to the image. It is apparent that manual thresholding provides the best result. The main reason for the failure of automatic thresholding methods is that they work directly on histogram information and they do not consider the shape information of the segmented image. However, even with manual thresholding, we should deal with noisy pixels that are segmented as part of the breast region. A human expert can easily separate the breast region from other parts with a high degree of accuracy, but, from a computational viewpoint, it is hard to exclude overlapping noisy regions from breast regions.

As a result, all thresholding methods that only consider histogram information can fail to properly segment the mammogram with accuracy. Applying preprocessing techniques to reduce the effect of noise or morphological operations on the resulting images does not guarantee the achievement of an accurate segmentation. In addition, due to the high degree of freedom in the breast shape, simple shape information cannot help thresholding methods to find the proper threshold value. Hence, we cannot expect to obtain accurate segmentation results by using segmentation techniques, and there is a high probability of finding smaller or larger breast regions using thresholding techniques.

**Figure 2:**
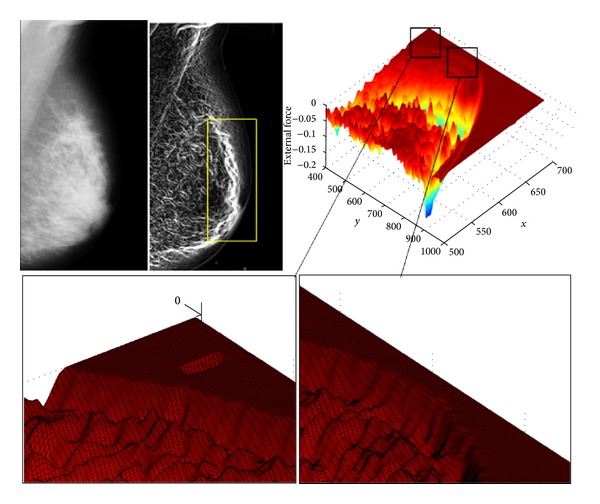
Importance of initialization in deformable models.

Technically, active contour models and level-set methods are applicable techniques for medical image segmentation, but they suffer from poor initialization. The main issue of those methods is that their accuracy depends on their initialization. In the case of mammograms, this kind of methods is usually initialized using thresholding techniques. As a result, they are vulnerable to remaining stuck in local optima rather than in the actual boundary.  Figure 2 illustrates this problem: the two graphs at the bottom of the figure show a small portion of the external force surface containing lots of local minima. It is apparent from these images that, if the algorithm is initialized a few pixels away from the correct minimum, the resulting boundary will be settled in a local minimum that does not correspond with the actual boundary. 

 Due to the fact that body tissues are not uniform inside the breast and its boundary, they will be characterized as textured regions on the final image. In addition, in a texture region, the intensity of each pixel might be different from that of its surroundings, and, hence, each pixel will have a positive gradient. As a result, the final external force function will be highly nonlinear and will contain lots of local minima.

**Figure 3:**
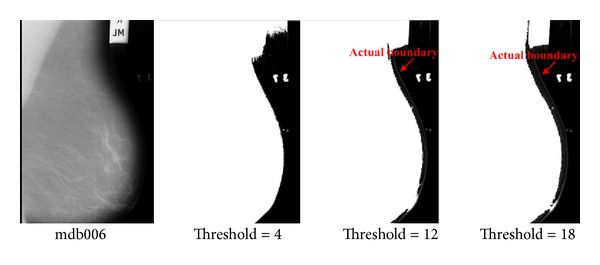
Example of interference of an artifact with the breast region.

In addition to the aforementioned problem, artifacts can also interfere and make deformable models fail. This is shown in Figure 3, where the mammogram is manually thresholded using values of 4, 12, and 18.

**Figure 4:**
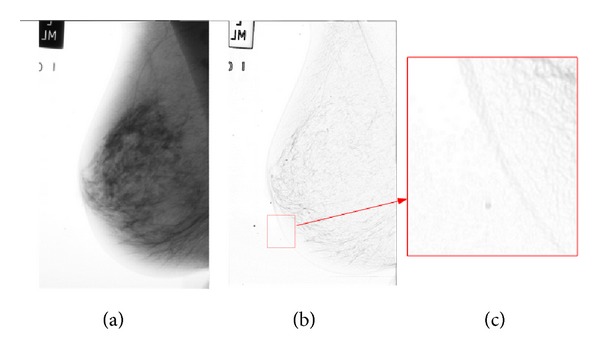
(a) Breast image, (b) edge map, and (c) a small portion of edge map.

As we stated before, body tissues are not uniform, and, as a result, they are characterized as textured regions. Hence, simple edge detection methods are not able to extract the boundary of the breast.  Figure 4 illustrates this fact.

Figure 4 shows that the breast image has a specific texture in its boundary. However, making a decision about which pixel belongs to the boundary is not simple since neighbor pixels do not have a uniform gradient. In addition to their textures, breast boundaries are characterized by smooth curves. Hence, the set of pixels that are extracted as boundary (in the rest of this paper, we refer to *breast boundary* or *boundary* indistinctly) pixels must lie on a smooth curve.

**Figure 5:**
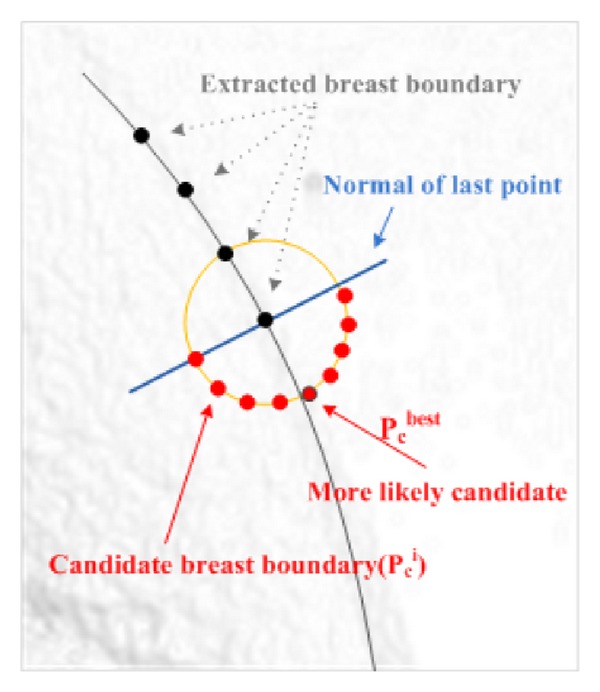
Candidates to be the next boundary points.

To start introducing our ideas, let us suppose that we are given the location of the initial point *P* on the boundary on the image. There is a set *C* = {*P*_*c*_^1^, *P*_*c*_^2^,…, *P*_*c*_^*N*^} of *N* points near *P* which are considered as candidates to be the next boundary point. Assuming that each candidate point has a specific texture on its surrounding, we can create another set *T* = {*T* ^1^, *T* ^2^,…, *T* ^*N*^} with *N* texture features corresponding to each candidate point. There is only one point in *C* which is more likely to be the next boundary point. As it is shown in Figure 5, point *P*_*c*_^*i*^ in set *C* is assumed to be more likely if its underlying texture has a high probability of being a boundary, and, in addition, it keeps the boundary smooth. The reason for selecting point *P*_*c*_^best^ as the most likely point is that it satisfies both criteria. In other words, there may be some points with high texture likelihood in set *C*. In this situation, the point that keeps the boundary smoother will be selected. From a probabilistic view-point, we are looking for a point within set *C* which maximises the following joint probability density function:

(1) $$\begin{array}{c} P_{c}^{\text{best}}=\underset{P_{c}^{i}\in C,T^{i}\in T}{\max}P\left(T^{i},P_{c}^{i},P^{k},\ldots ,P^{1}\right), \end{array}$$

where *T* ^*i*^ refers to the observed texture feature surrounding,  *P*_*c*_^*i*^, *P* ^*k*^,…, *P* ^1^  accounts for the smoothness of the boundary, and *P*_*c*_^*i*^ is the current candidate pixel. If we could find a model for the above joint probability distribution, we could also easily find the most likely candidate. Notwithstanding, finding such probability model is a hard problem.

With the aim to simplify this model and to make it tractable, we assume that the smoothness is a second-order Markov process. Applying this assumption to (1), we obtain the following equation:

(2) $$\begin{array}{c} P\left(T^{i},P_{c}^{i},P^{k},\ldots ,P^{1}\right)=P\left(T^{i},P_{c}^{i},P^{k},P^{k-1}\right). \end{array}$$

Using the product rule of probability, we can factorize (2) as follows:

(3) P(Ti,Pci,Pk,…,P1)=P(Ti ∣ Pci,Pk,Pk−1)×P(Pci ∣ Pk,Pk−1)×P(Pk ∣ Pk−1)×P(Pk−1).

Assuming that *T* ^*i*^ and *P*_*c*_^*i*^ are conditionally independent given *P* ^*k*^ and *P* ^*k*−1^ and considering that *T* ^*i*^ and *P*_*c*_^*i*^ are independent variables, we can rewrite (3) as follows:

(4) P(Ti,Pci,Pk,…,P1)  =P(Ti)×P(Pci ∣ Pk,Pk−1)×P(Pk ∣ Pk−1)×P(Pk−1).

In (4), *P*(*T* ^*i*^) is the probability of the texture in the region surrounding point *P*_*c*_^*i*^ to be part of the boundary, and *P*(*P*_*c*_^*i*^ | *P* ^*k*^, *P* ^*k*−1^) accounts for the smoothness and computes the location probability of a candidate point *P*_*c*_^*i*^ given the locations of two previously detected boundary points. In addition, *P*(*P* ^*k*^ | *P* ^*k*−1^) is the probability of selecting the boundary point *P* ^*k*^ given *P* ^*k*−1^, and *P*(*P* ^*k*−1^) indicates the prior location probability of the second previously detected boundary point. Without loss of generality, we can ignore the last two terms in (4) since they are constant and do not affect the determination of *P*_*c*_^*i*^ and *T* ^*i*^. Hence, the final joint probability can be expressed as follows:

(5) $$\begin{array}{c} P\left(T^{i},P_{c}^{i},P^{k},\ldots ,P^{1}\right)=P\left(T^{i}\right)\times P\left(P_{c}^{i}\;|\;P^{k},P^{k-1}\right). \end{array}$$

In a nutshell, to trace the boundary of the breast, we just need to find the probability distribution of the texture features, *P*(*T* ^*i*^), as well as the probability distribution of the candidate point given the last two boundary points, *P*(*P*_*c*_^*i*^ | *P* ^*k*^, *P* ^*k*−1^). In the following sections, we describe how to find these probability distributions.

### 3.1. The Texture Probability Model

*P*(*T* ^*i*^) represents the probability of the texture of the candidate point *P*_*c*_^*i*^ being part of the boundary. To define this probability model, we need some training mammograms in which the breast boundary is manually identified. Those mammograms are used as the ground truth for boundary detection. Then, for each point *P*_*c*_^*i*^ on the ground-truth boundary, its surrounding texture patch is selected, and a feature extraction algorithm is applied on it. After this procedure is repeated for all boundary points of all training images, we obtain a collection of texture feature vectors *F* for all boundary points. Finally, we find a probability model for the collection *F*. To this end, we determine the feature extraction algorithm and the probability distribution model. In the following sections, we provide more details about this procedure.

#### 3.1.1. Texture Features

There are lots of efficient texture feature extraction algorithms such as first-order statistical measures, cooccurrence matrix, autocorrelation, Voronoi tessellations, Markov random fields, fractals, Fourier transforms, discrete cosine transforms, and Gabor filters banks [33, 34]. To describe a texture patch in a mammogram efficiently, we need an algorithm that considers the order of occurrence of pixel intensities relative to other pixels or a fixed value. Ojala et al. [35] introduced the *local binary pattern* (LBP) in 1996 and then extended it in 2002 [36]. Since then, LBP features are extensively used in machine vision problems. In addition to their computational efficiency, they also have excellent discriminative power which makes them suitable for classification problems [37–39].

To extract the feature vector of a given texture patch, we compute the LBP value of each pixel in the texture patch. Suppose that *z*_0_ is the intensity of the pixel we want to calculate its LBP value. *z*_1_ ⋯ *z*_8_ are the intensities of the pixels in its 8-neighborhood. The LBP value of the pixel *z*_0_ is computed as follows:

(6) $$\begin{array}{c} \text{LBP}\left(z_{0}\right)=\overset{8}{\underset{i=1}{\sum }}2^{i-1}\delta \left(z_{i}-z_{0}\right). \end{array}$$

**Figure 6:**
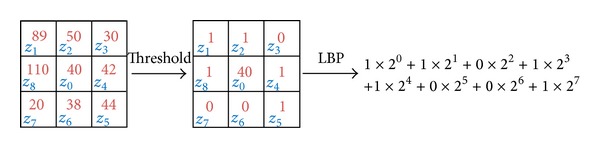
Computation of the LBP value for a pixel using its 8 neighbors.

In (6), *δ*(*u*) is 1 if *u* ≥ 1 and 0 otherwise. In fact, the above calculation leads to an 8-bit value for each pixel.  Figure 6 illustrates this procedure. 

**Figure 7:**
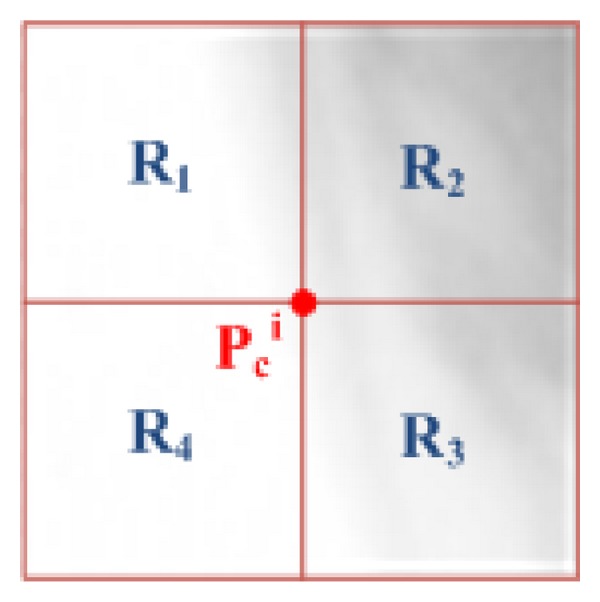
Division of the image patch into four regions for the extraction of texture features with LBP.

 After computing the LBP values of all pixels in a given patch, a histogram of LBP values is calculated. This histogram is used as the feature vector for that patch. In this paper, to extract the vector of features from a patch, we propose to divide it into four equal subregions as shown in Figure 7. Considering this division shown in Figure 7, an LBP histogram of each subregion is computed and concatenated into a single vector so as to build the feature vector of the underlying patch of the candidate pixel *P*_*c*_^*i*^.

#### 3.1.2. Probability Modeling

 After collecting the feature vectors of the boundary pixels, we should find a way to model their distribution. Usually, a Gaussian mixture model (GMM) is used in these cases. However, as we show in the experimental results, GMM is not able to model their distribution accurately. One of the difficulties in GMM is the determination of the number of components of the model. In addition, expectation maximization algorithms can get stuck in local minima, and, consequently, GMM cannot model the distribution accurately.

In this paper, we use support vector machines (SVMs) to calculate the probability of the feature vectors. The intuition behind this approach is as follows: we suppose that a feature vector can be classified into two classes, namely, “boundary” and “nonboundary.” Obviously, there is a decision boundary which separates these two classes. The intuition behind calculating the probability using support vector machines is that the probability of the feature vectors near the decision boundary will be close, and, actually, on the decision boundary, the probability is equal to 0.5. Also, inside each region, the probability is relative to the distance of the feature vector to the decision boundary. Technically, the decision function of a support vector machine is calculated as follows:

(7) $$\begin{array}{c} f\left(x\right)=\overset{m}{\underset{i=1}{\sum }}\alpha_{i}e^{-\gamma ||x_{i}-x{||}^{2}}+b. \end{array}$$

In this equation, *m* is the number of support vectors, and *x*_*i*_ is the center of the *i*th kernel. *α*_*i*_ and *b* are the parameters to be found during the training process. Thus, given two classes of data, our goal is to estimate the following:

(8) $$\begin{array}{c} p\left(y=i\;|\;x\right),\;i=1,2. \end{array}$$

To this end, Wu et al. [40] used (7) to estimate the class probabilities, *r*_*ij*_ and optimize the following function to obtain the values of *p*_*i*_ the following:

(9) minp 12∑i=12 ∑j;j≠i(rijpi−rjipj)2subject  to pi≥0, ∀i, ∑i=12pi=1.

In this paper, we use the same approach to calculate the probability of the feature vectors. In a nutshell, we collect the feature vectors of boundary points as well as the feature vectors of nonboundary points from the training mammograms so as to train a two-class SVM. Then, for a new feature vector *x*, we use the above method to calculate its class conditional probability.

### 3.2. Smoothness Probability

To calculate *P*(*P*_*c*_^*i*^ | *P* ^*k*^, *P* ^*k*−1^), we collect the following information from each training boundary:

(10) slopek=[atan 2(Pxk−Pxk−1,Pyk−Pyk−1),atan 2(Pxk−1−Pxk−2,Pyk−1−Pyk−2)].

**Figure 8:**
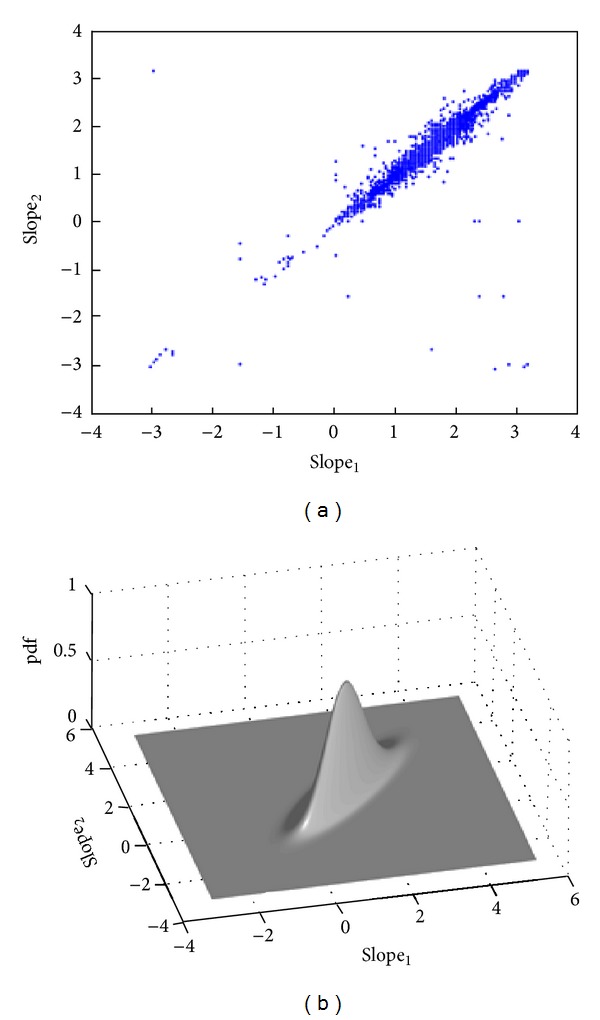
Distribution of the vector slope_*k*_ in a 2D Euclidean space.

The vector slope_*k*_ indicates the relative slope between the current point and the last two boundary points. We collect these vectors for all training ground-truth boundaries.  Figure 8 shows the plot of these vectors on a 2D coordinates system. It is apparent from this figure that the distribution of the vector slope_*k*_ follows a Gaussian distribution model. Hence, we can calculate *P*(*P*_*c*_^*i*^ | *P* ^*k*^, *P* ^*k*−1^) using the following:

(11) $$\begin{array}{c} P\left(P_{c}^{i}\;|\;P^{k},P^{k-1}\right)=\frac{1}{\sqrt{2\pi \left|\Sigma \right|}}e^{\left(-1/2){\left(x-\mu \right)}^{T}\Sigma^{-1}(x-\mu \right)}, \end{array}$$

where *μ* is the mean vector and Σ is the covariance matrix of the *i*th component.

### 3.3. Prior Probability of the Location

**Figure 9:**
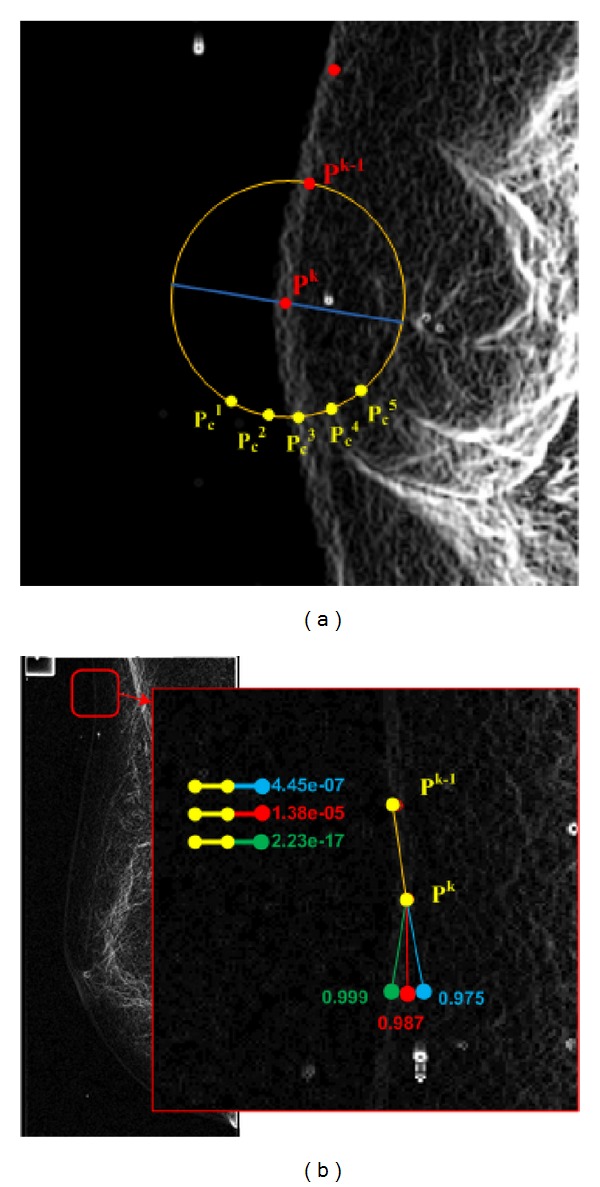
The need for prior knowledge.

Prior probability is important as we illustrate in Figure 9, where a small portion of a breast image is shown. Let us suppose that the algorithm has traced the boundary and has reached point *P* ^*k*^. Now, the algorithm proceeds by finding the best next candidate. Amongst the different candidates in the radius *r* of point *P* ^*k*^, the five points with more likelihood are shown in Figure 9 (labeled as *p*_*c*_^1^ ⋯ *p*_*c*_^5^). Each of these candidates has a probability to be a breast boundary pixel, but, amongst them, *P*_*c*_^2^, *P*_*c*_^3^, and *P*_*c*_^4^ have higher likelihood according to the texture probability model, and, obviously, *P*_*c*_^3^ has the highest likelihood.

Considering the line segments between (*P* ^*k*−1^, *P* ^*k*^, *P*_*c*_^2^) and the line segments between (*P* ^*k*−1^, *P* ^*k*^, *P*_*c*_^3^), we realize that by selecting *P*_*c*_^2^ the boundary would be smoother than by selecting point *P*_*c*_^3^. Also, if we consider all training boundary segments, the probability of occurrence of the (*P* ^*k*−1^, *P* ^*k*^, *P*_*c*_^3^) segment would be lower than (*P* ^*k*−1^, *P* ^*k*^, *P*_*c*_^2^). Hence, the result of multiplying the texture probability of *P*_*c*_^2^ and (*P* ^*k*−1^, *P* ^*k*^, *P*_*c*_^2^) would be greater than the result of multiplying the texture probability of *P*_*c*_^3^ and (*P* ^*k*−1^, *P* ^*k*^, *P*_*c*_^3^). As a result, *P*_*c*_^2^ would be selected as the next boundary pixel.

Mathematically, in a support-vector-machine-based probability estimation, there is a direct relation between the distance of the feature vector from the support vectors and its probability. However, since the core of the probability estimation in the support vector machine is a *sigmoid* function, we can expect that there is no sharp transition at high probabilities. As it is indicated in Figure 9, three sample points have close probabilities while the smoothness probability, of the green point is much lower than the smoothness probability of the red and blue points. Although the green point is the best candidate for the boundary, by considering the probabilities in (5), the red point would be selected as the next boundary point.

**Figure 10:**
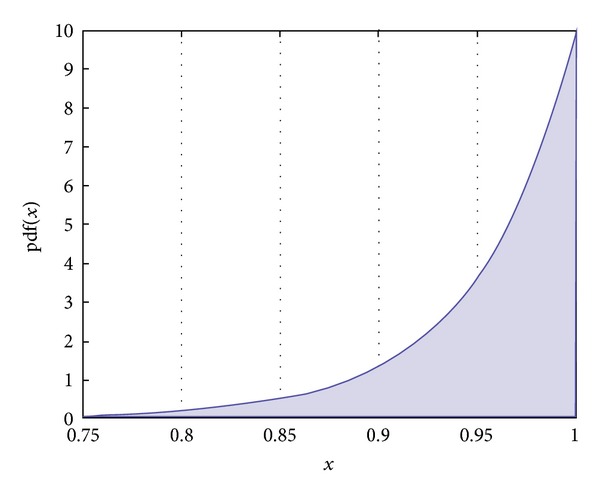
The Laplace distribution with *μ* = 1 and *β* = 0.05.

To avert this problem, we need a prior probability to penalize points such as *P*_*c*_^2^. As we mentioned above, the problem appears because of the smooth probability transition between *P*_*c*_^2^ and *P*_*c*_^3^. By putting these together, we add a penalizing factor to the texture probability model *p*(*T*) so as to sharpen its density function. In this study, we have used the Laplace distribution since it has sharper transition even in the high probabilities. This is shown in Figure 10. Generally, the Laplace distribution is a piecewise function, but, in our application, we have just selected the lower function as follows:

(12) $$\begin{array}{c} P\left(P_{c}^{i}\right)=\text{Laplace}\left(x;\mu ,\beta \right)=\frac{e^{-(-(\mu -x)/\beta )}}{2\beta }. \end{array}$$

In (12), *x* is equal to *P*(*T*). The interpretation of this equation is that if the texture probability is high it is much more likely for the point to be a boundary point. If we apply this prior probability on the candidate points depicted in Figure 9, point *P*_*c*_^3^ would be selected instead of point *P*_*c*_^2^. Considering the penalizing factor, the original (5) is changed to the following equation:

(13) $$\begin{array}{c} P\left(T^{i},P_{c}^{i},P^{k},\ldots ,P^{1}\right)=P\left(T^{i}\right)\times P\left(P_{c}^{i}\;|\;P^{k},P^{k-1}\right)\times P\left(P_{c}^{i}\right). \end{array}$$

### 3.4. Initializing the Algorithm

In our algorithm, initializing means finding the starting point of the boundary from which we start the analysis. Since this is the first point, there is no information about previous points, and the smoothness cannot be computed (i.e., *P* ^*k*^ and *P* ^*k*−1^ in (13) are unknown). To deal with this situation, we use marginalization as follows:

(14) $$\begin{array}{c} P\left(T^{i},P_{c}^{i}\right)=\int_{P^{k}}\int_{P^{k-1}}P\left(T^{i}\right)\times P\left(P_{c}^{i}\;|\;P^{k},P^{k-1}\right)\times P\left(P_{c}^{i}\right). \end{array}$$

Marginalization over *P* ^*k*^ and *P* ^*k*−1^ is equivalent to estimating the joint probability of (13) regardless of the values of these two variables. Also, finding the pixel with the maximum value for (14) implies that the underlying texture of that pixel must correspond to the boundary class. In addition, notice that the term *P*(*P*_*c*_^*i*^) in (14) does not affect the final result. Consequently, to initialize our algorithm, the input image is scanned from top to bottom and from left to right. Each pixel at a time is analyzed so as to determine its class (i.e., boundary or non boundary). Once a pixel is found to belong to the boundary class, all of the pixels in that row are also analyzed, and that with maximum probability is selected as the initial point for the algorithm to start.

### 3.5. Tracing the Breast Boundary

Let us suppose that we have trained the texture probability model *P*(*T*) as well as the smoothness probability model *P*(*P*_*c*_^*i*^ | *P* ^*k*^, *P* ^*k*−1^). To find the starting point of the boundary, pixels of the mammogram are scanned from left to right, and, for each pixel *P*_mn_, a window of size *w* centered on *P*_mn_ is considered. The pixels inside this window are selected, and the LBP feature vector is extracted for these pixels.

**Figure 11:**
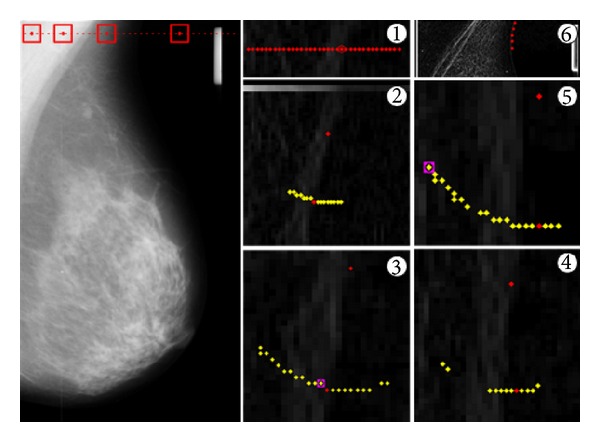
Tracing the boundary.

Then, the class of this feature vector is determined using the trained SVM, and if it is classified as a “boundary” point, its probability is computed using the same SVM. This process is repeated for every pixel of the same row of the image. If any boundary point is found, the one with maximum probability is selected as the starting point of the boundary. As it is shown in the first step of Figure 11, the pixel with a red circle around it is selected as the starting point. 

To find the second point of the boundary, all pixels in a radius *r* from the starting point are considered as candidate boundary points. Again, the candidate points are classified, and the probability of those classified as “boundary” is computed. Then, (13) is evaluated on these pixels, and the one with maximum value is selected as the second boundary point. Step 2 in Figure 11 shows this process. The yellow dots are the candidate points classified as “boundary,” and the red dot is the one with maximum value for (13). The process of selecting candidate points in a radius *r* from the last selected boundary point, the computation of the probability of those classified as “boundary,” and the selection of the pixel with the maximum value for (13) are repeated until no more points are found.

The important role of the smoothness factor is emphasised in steps 3 and 5 of Figure 11. In addition to the red dots, there are other pixels with a magenta square around them. These are the pixels which would be selected by using texture probabilities only. Since finding a perfect probability model is a very hard task, we can expect that, in some cases, the SVM computes the probability inaccurately. However, even if the texture is computed accurately, we might force the boundary to be smooth. At step 3, as expected, the texture probability of the magenta pixel is higher than that of the red pixel, but if the magenta pixel is selected, the smoothness of the boundary is lower. Hence, when the smoothness factor is considered, the red pixel is selected as the next boundary point.

However, the texture probability of the magenta pixel at step 5 is computed inaccurately (which may be caused by the feature vector or the SVM itself). Again, selecting this pixel lowers the smoothness of the boundary, and, as a result, the red pixel is selected as the next boundary point.

## 4. Experiments

We have implemented the proposed method in the MATLAB 2010 environment. In addition, we used LibSVM [41] to train the SVM classifier. The probability estimation algorithm for the support vector machine is implemented within this library. To avoid overfitting problems, we have used 5-fold crossvalidation during the training phase. Also, to find the best values for the parameters of the SVM, we have utilized a logarithmic grid search together with a 5-fold cross-validation.

In the following experiments, the texture feature vectors of both breast and pectoral muscle pixels are extracted in windows of size 50 × 50 pixels, and the candidate points are computed within a radius of *r* = 20 pixels from the current pixel. Finally, the values of the parameters *μ* and *β* in the Laplace distribution were selected as 1 and 0.05, respectively.

With the aim to reduce the dimensionality of the feature vectors, we applied *principal component analysis* (PCA) on the collected feature vectors. Also, the number of selected PCA basis vectors has been computed automatically by dividing the eigenvalues by the sum of all eigenvalues and then finding the index in which the cumulative sum of this division is greater than 0.99.

### 4.1. Mini-MIAS Database

 The Mammographic Image Analysis Society (MIAS) has generated a digital database of mammograms. Films taken from the UK National Breast Screening Programme have been digitized to 50-micron pixel edge with a Joyce-Loebl scanning microdensitometer, a device linear in the optical density range 0–3.2 representing each pixel with an 8-bit word [42].

For our experiments, we have used the mini-MIAS [42] database. This database contains 322 mammograms which are obtained by digitizing the original MIAS database. The database has been reduced to 200-micron pixel edge and clipped/padded so that every image is 1024 × 1024 pixels, and it is publicly available for scientific research at the site of the University of Essex.

In order to manually extract the breast boundary and prepare training data, the gradients of sample images were processed using Adobe Photoshop CS, and the boundary of the breast was manually determined using a combination of threshold and magnetic and polygonal lasso tools. Also, in some cases, we used an interactive threshold method to find the ground-truth boundaries. By this way, we selected 57 images and extracted their boundaries manually.

The selection of the training images was done manually such that they cover different shapes and textures as much as possible. For example, based on the size of the breast, the shape of the mammogram can be near-flat, semicurved, or highly curved. From the texture perspective, some mammograms are noisy in their boundaries. In addition, some of them have dense tissues near the nipple area. In summary, the selected 57 training images provide a convenient variety of shape and texture information.

It is clear that different radiologists can draw the boundaries of the same mammogram differently. In addition, a radiologist may extract different boundaries for the same mammogram at different times. Hence, there is not a perfect or exact ground-truth boundary for a mammogram. From a probabilistic point of view, there are uncertainties in the boundary. Notwithstanding, it should be noted that our algorithm is based on probability by learning the texture and the shape of the training boundaries and computing their corresponding probability density functions. Hence, even if there are different boundaries for a typical mammogram, however, there is just one more-likely boundary which is extracted using our algorithm. As a result, our algorithm does not require perfect or identical training ground-truth boundaries. Using the extracted training boundaries, positive and negative samples are collected to train the SVM as follows: for every boundary pixel, the pixel and its left and right pixels were selected as positive samples. Also, for each mammogram, 900 random pixels on the image are selected as negative samples. The negative samples which were close to the boundary were discarded. Positive and negative samples were collected from all training images, and, in total, 50176 negative samples and 37704 positive samples were obtained. Finally, we selected another 37 hard-to-process test images from the database and extracted their ground-truth boundaries in the same way. The test boundaries are not used for the training of the SVM and the smoothness probability model. We have selected only 37 test images for two main reasons: first, it is time consuming to extract the ground-truth boundary of the complete set of images in the database, and, second, in most cases, the texture and the shape information of the mammograms are similar, so they cannot be good test cases.

### 4.2. Accuracy Measure

Each boundary is represented by a set of points. Given the extracted boundary *P*_*e*_ = {*p*_*e*_^1^, *p*_*e*_^2^,…, *p*_*e*_^*m*^} and the ground-truth boundary *P*_*g*_ = {*p*_*g*_^1^, *p*_*g*_^2^,…, *p*_*g*_^*n*^} (where *m* could be different from *n*), we want to know how close are *P*_*e*_ and *P*_*g*_. In other words, we want to measure the accuracy of the extracted boundary in comparison with the ground-truth boundary.

**Figure 12:**
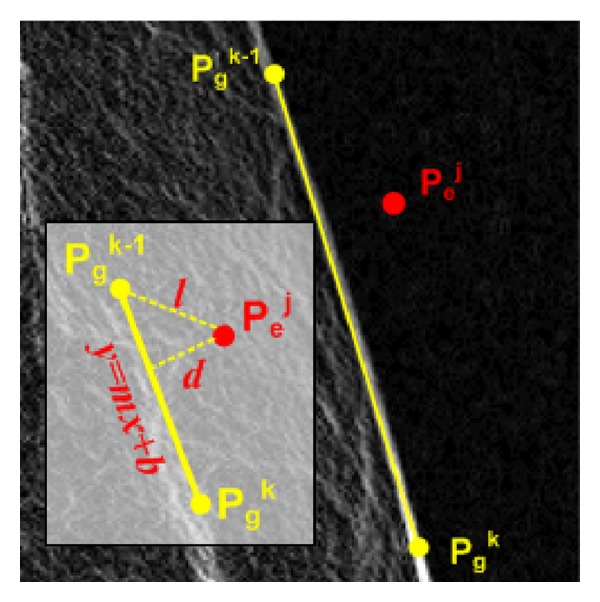
Estimating the accuracy of a boundary on the extracted boundary.

Let us suppose that the ground-truth boundary points are *p*_*g*_^*k*−1^ = (*x*_*g*_^*k*−1^, *y*_*g*_^*k*−1^) and *p*_*g*_^*k*^ = (*x*_*g*_^*k*^, *y*_*g*_^*k*^) and that the extracted boundary point is *p*_*e*_^*j*^ = (*x*_*e*_^*j*^, *y*_*e*_^*j*^) as shown in Figure 12. The line passing through points *p*_*g*_^*k*−1^ and *p*_*g*_^*k*^ is *y* = *f*(*x*) = *mx* + *b*, where *m* is the slope of the line and *b* is the vertical distance from the origin. As the figure shows, the distance *d* is the actual difference between the two boundaries.

To compute the value of *d*, we can use the following equation:

(15) d(pej,pgk,pgk−1)=|yej−mxej−b|m2+1,  m=ygk−ygk−1xgk−xgk−1,  b=−mxgk+ygk.

Now, we can use (15) to estimate the accuracy of *P*_*e*_ using *P*_*g*_:

(16) accmean=1m∑j=1md(pej,pgk,pgk−1),  pgk=arg min⁡p∈Pg||p−pej||,  pgk−1=arg min⁡p∈Pg,p≠pgk||p−pej||.

In order to estimate the accuracy of the extracted boundary, for each extracted boundary point *p*_*e*_^*j*^, the two closest points in *P*_*g*_ are found. Then, the accuracy of *p*_*e*_^*j*^ is computed using (15). The average of these values is used as the accuracy of the extracted boundary.

### 4.3. Curve Measure

 In addition to the accuracy, the extracted boundary has to be smooth. The smoothness of the curve can be formulated by its first and second derivatives. For a point *p*_*e*_^*j*^ on the extracted boundary, the first and second derivatives are defined as follows:

(17) $$\begin{array}{c} {\left|\delta p_{e}^{j}\right|}^{2}={\left(x_{e}^{j}-x_{e}^{j-1}\right)}^{2}+{\left(y_{e}^{j}-y_{e}^{j-1}\right)}^{2},\\ {\left|\delta \delta p_{e}^{j}\right|}^{2}={\left(x_{e}^{j-1}-2x_{e}^{j}+x_{e}^{j+1}\right)}^{2}+{\left(y_{e}^{j-1}-2y_{e}^{j}+y_{e}^{j+1}\right)}^{2}. \end{array}$$

The first derivative measures the stretchiness, and the second derivative measures the curvature. Using (17), the curve measure of the boundary *P* is calculated as follows:

(18) $$\begin{array}{c} C\left(P\right)=\overset{m-1}{\underset{2}{\sum }}{\left|\delta p^{j}\right|}^{2}+{\left|\delta \delta p^{j}\right|}^{2}, \end{array}$$

where *m* is the total number of boundary points. Finally, to compare the extracted boundary *P*_*e*_ with the ground-truth boundary *P*_*g*_, we can measure their curve values using (18) separately and calculate their absolute difference:

(19) $$\begin{array}{c} \text{curve}=\left|C\left(P_{e}\right)-C\left(P_{g}\right)\right|. \end{array}$$

### 4.4. Evaluation of Breast Boundary Extraction

**Figure 13:**
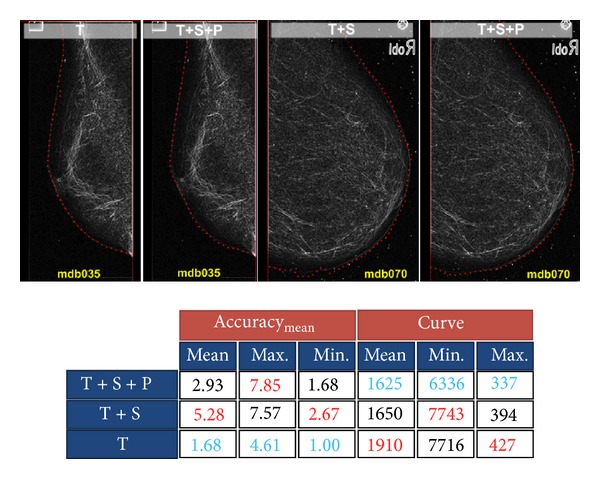
Result of tracing the boundary of the breast.

With the aim to assess the performance of our algorithm, we have run it three times with different configurations. In the first configuration, all terms in (14) were considered. In other words, the boundary of a typical mammogram was extracted considering texture, smoothness, and prior probabilities. Then, the prior probability factor was discarded, and the algorithm was applied on the test images, again. Finally, in the third configuration, both the prior and the smoothness probabilities were ignored, and the boundary was extracted using texture information only. For each extracted boundary, their accuracy and curve measures were computed using (16) and (19), respectively.  Figure 13 shows the results.

 In Figure 13, red and blue numbers indicate the minimum and maximum values of each column, respectively. Also, *T*,  *S*, and  *P*  are abbreviations for *texture*, *smoothness*, and *prior* probabilities, respectively. As the figure shows, the minimum acc_mean_ is obtained when the boundary is extracted using texture information only. In addition, the average absolute difference between the curve measure of the extracted boundary and the ground-truth boundary is maximum when we use texture information only.

Although we are interested in low values of acc_mean_, we should also pay special attention to the values of the curve measure. High values of the curve measure indicate that the extracted boundary is jittery, while low values indicate smooth boundaries. Considering just the value of acc_mean_ could be seen as a greedy decision. According to this value, the boundary extraction using just the texture information is more accurate than the algorithm in which we use smoothness and prior probabilities as well.

As the two left images in Figure 13 show, the extracted boundary inside the yellow square region is completely inaccurate when we only utilize texture information. However, when we add smoothness and prior probabilities, a more accurate boundary can be extracted. This problem originates from the fact that the trained probability model is not perfect and it can produce wrong probabilities in some cases. One obvious solution to this problem is to collect more training data, but we should note that a larger dataset might increase the number of support vectors, and, consequently, it increases the complexity of the probability model. In addition, larger datasets do not guarantee a perfect model either. We have decided to cope with this problem by using the smoothness probability.

According to the table under Figure 13, discarding the prior probability decreases the accuracy of the extracted boundary significantly. This is also shown in the two right images of Figure 13 in which the boundary inside the blue region is completely inaccurate when we have discarded the prior probability. As we mentioned before, this problem appears because the core of the probability estimation is a logistic function which does not have sharp transitions for high probabilities. To tackle this problem, we have added an extra factor to our model to put more weight on higher probabilities. Our algorithm proves to be reliable and accurate when we use texture, smoothness, and prior probabilities together.

There are only a few parameters in our algorithm that are determined manually. The first parameter is the size of the window in which texture features are extracted. Although we have used a predefined value for this parameter, it can be defined as a function of the size of the mammogram. Also, it is possible to use multiscale window sizes with different SVMs and select the candidate point using voting algorithms. The second parameter is the distance between candidate points which can be a function of the size of the mammogram or a fixed value. The only parameter that can affect the result of the algorithm is the *β* value in the Laplace distribution. Note that, once the value of *β* is determined, there is no need to change it for different mammograms.

Generally, the tissues of the breast are categorized as *fatty* or *dense*. However, since we consider the tissues on the boundary of the breast as a separate type of tissues, in this paper, we have categorized them into *fatty*, *dense*, and *boundary*. Bearing this in mind, our algorithm discards the fatty and dense tissues using SVM and just accepts those which are classified as boundary tissues. Hence, different types of tissues do not affect the boundary extraction process.

Among different approaches, image-processing-based methods can be used in real-time applications, but it should be noted that they do not guarantee to find an accurate boundary. On the other hand, deformable models are slower than image-based methods, and their time complexity directly depends on the number of points that are used to form the contour. Although, they are more accurate than image based methods they can fail to find the boundaries especially when artifacts such as labels are connected/overlapped with the breast.

The time complexity of our method is higher than those of two previous methods since, instead of using simple rules for selecting a pixel as boundary or nonboundary, our method uses machine-learning techniques to classify the pixels using their texture information. This increases the localization accuracy and stability of the method significantly.

Notwithstanding, a high degree of accuracy and reliability comes with a high time complexity. The most time-consuming part of our algorithm is classifying and calculating the probability of candidate points using SVM. The complexity of SVM depends on the number of support vectors, and the number of support vectors depends on the degree of nonlinearity of the feature space. The key to increase the speed of the algorithm resides in replacing the SVM by other probabilistic methods. If we can achieve this goal, the time complexity of our algorithm could be decreased as much as those of deformable models.

### 4.5. Evaluation of Pectoral Muscle Boundary Extraction

**Figure 14:**
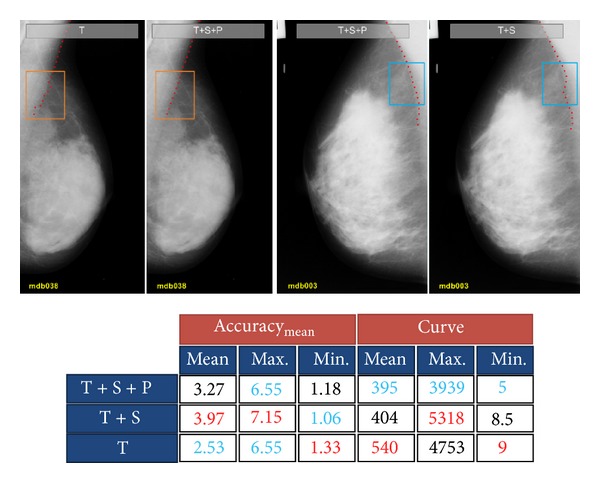
Result of tracing the boundary of the pectoral muscle region.

In order to extract the boundary of pectoral muscles, we used the same probabilistic approach that we used for the boundary of the breast except for the probability density functions that are modeled by using training data from the pectoral muscle boundary. Again, to evaluate the performance of the proposed algorithm, we applied the method in three different configurations as in the previous experiment on 38 test images. The result is shown in Figure 14.

As Figure 14 shows, the minimum acc_mean_ is obtained when the boundary is extracted only using texture information. In addition, the average of the absolute difference between the curve measure of the extracted pectoral muscle boundary and the ground-truth boundary is maximum when we use texture information only. However, the performance of the algorithm with texture, shape, and prior information is numerically close to the case in which we have just used the texture information. On the other hand, discarding the prior probability causes inaccurate results.

To visually analyze the results, two representative images were selected. It is apparent from the two left images in Figure 14 that the pectoral muscle boundary inside the orange rectangle region is inaccurate when the shape and prior information are discarded. This happens because when this information is ignored the algorithm selects the pixels in which their texture probability is the highest. This can produce good results whenever the texture probability density function is perfectly modeled using training feature vectors. However, if the training data is not sufficient or accurate, the uncertainty of the model increases, and, consequently, pixels may be selected inaccurately.

On the contrary, the shape information adds a constraint to the probability model, and, in addition, prior probability makes fast nonlinear transitions on the final density function. This prevents the algorithm from selecting wrong candidate points. As in the previous experiment, prior information guides the algorithm to put more value on the pixels with higher texture probabilities, and, for the same reasons that we discussed previously, using this information, the extracted pectoral muscle boundary is more accurate.

### 4.6. Failure Analysis

**Figure 15:**
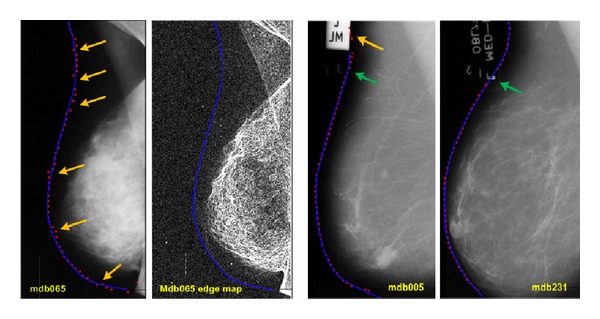
Failure analysis of the algorithm.

 Although our method is able to extract the boundary of the breast and pectoral muscle accurately, there are still cases in which the algorithm makes wrong decisions in selecting the next candidate points. This problem appears in the presence of significant amounts of noise that distorts the boundary of the breast. Also, the overlap of artifacts with the breast boundary might lead to wrong decisions. These issues are shown in Figure 15.

 This figure shows three different images from mini-MIAS database. The blue curves are the ground-truth boundaries of the breast, and the red dots are the ones extracted by using our algorithm. In the two images on the left, the extracted boundary of the image “mdb065” and its corresponding edge map are shown. There are some yellow arrows on the image pointing to those parts of the boundary which are selected inappropriately. To find out the reason of these wrong selections, we might refer to edge map of the mammogram (on the right). According to the edge map, there is a significant amount of noise on the image which has highly distorted the actual boundary. Also, the algorithm relies on extracted LBP features, but, due to the noise, uncertain features are extracted for candidate points, and, consequently, the texture probability is estimated inaccurately. As result, the candidate points are selected wrongly in some parts of the boundary.

The two images on the right of Figure 15 show that the artifacts are overlapped with the boundary of the breast. The yellow arrow points to the section of the boundary that is wrongly selected and the green arrows point to those sections that are properly selected, regardless of the artifacts. Again, the wrong candidate point selection is due to the extracted feature vector.

Since we are using a probabilistic framework, we should be able to deal with uncertainties. From a probabilistic perspective, the pixels of the image with inaccurate feature vectors must have lower probabilities to be candidate points. However, if the probability density function of the feature vectors is not accurately modeled or if the feature vectors of both a noisy image patch and an ideal image patch are placed in the same region of feature vector space, their probabilities are computed wrongly, and inappropriate candidates are selected as boundary points.

### 4.7. LBP versus HOG and Statistics

**Figure 16:**
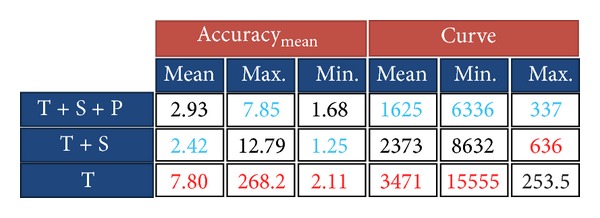
Comparison between LBP, HOG, and first-order statistical features.

As we stated before, the LBP is a powerful and efficient feature extraction method with high discriminative power. In this experiment, we utilize different feature extraction methods and compare them with LBP features. As with LBP, the dimensionality of the feature vector is reduced by applying PCA. In this experiment, we have used histogram of oriented gradients (HOG) and first-order statistical features. The cell size and block size of the HOG method are selected as 20 and 2, respectively. Also, a 9-bin histogram was used to extract the final feature vector. Regarding the statistical values, we used *mean*, *variance*, *skewness*, *kurtosis*, *maximum*, *minimum*, and *entropy*.  Figure 16 shows the results of this experiment. It is apparent that statistical features are not a good choice for this problem. On the other hand, although the average accuracy of the HOG method is better than with the LBP method, its curve measure is much higher than the LBP method. This results in a jittery boundary. As a result, the LBP method (used by our algorithm) proves to be a good choice for modeling the texture probability of the boundary points.

### 4.8. Comparisons and Discussion

**Figure 17:**
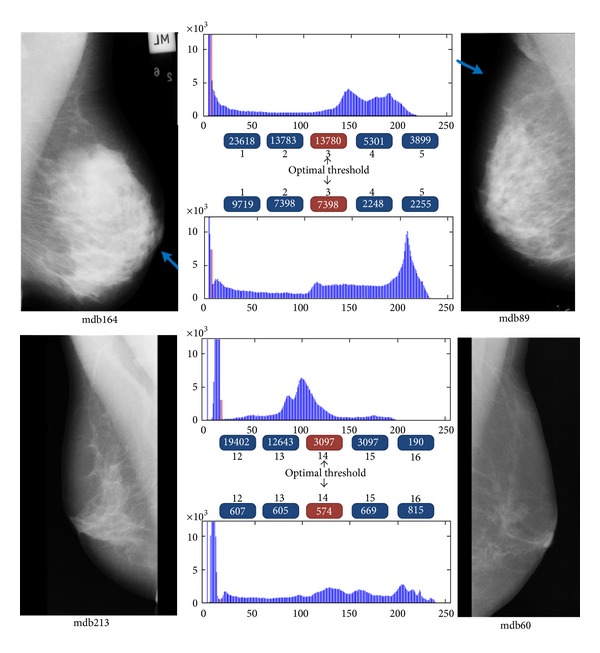
Optimal threshold values and histograms of four mammograms.

 As we mentioned in the previous sections, threshold-based algorithms and active contour models are the most widely used techniques for extracting the boundary of the breast. Both can fail to extract an accurate boundary even in simple mammograms. In this section, we elaborate on this claim. We start our discussion by referring to Figure 17. This figure shows four different mammograms from the mini-MIAS database. In an interactive procedure, we found their optimal threshold value. This is equal to gray levels for 3 and 14 for the mammograms at top and bottom of the figure, respectively. Automatic threshold selection methods such as the Otsu method and the maximum entropy thresholding can fail in finding the proper value. For example, consider the second histogram (counting from top). There are dominant peaks at intensities near gray-level 210. Again, let us assume that we have applied an automatic threshold selection method on the histogram and obtained gray-level three as the optimal threshold. Now, there is another mammogram with exactly the same histogram except for the fact that histogram has approximately a flat shape around gray-level 210.

Intuitively, we would expect the automatic threshold selection method to return the same gray-level (three) as threshold value, but, if we refer to the decision functions of automatic threshold selection methods, we realize that there is no guarantee that the gray-level remains the same for the second histogram as well. For this reason, most authors propose to select the threshold value using shape information of the histogram instead of statistical criteria.

However, these methods are not reliable, and they can fail to find the proper threshold. This is shown in Figure 17. There are 5 numbers near each histogram which indicate the gray-level value and its corresponding histogram value. For example, in the first histogram, the optimal threshold value is three (red rectangle), and there are 13,780 pixels in the mammogram with this gray-level. Beside the optimal threshold and its corresponding histogram value, we have also shown the histogram values for the two previous and next gray-levels.

In the first histogram, the optimal threshold value is not a local minimum nor is it a local maximum. In the second and third histograms, the optimal threshold value is located at a saddle point of the histogram. However, in the fourth histogram, the optimal threshold is a local minimum of the histogram.

Suppose that we specify that the optimal threshold value is located at a local minimum on the histogram. Since there can be many local minima in the histogram, the question is which local minimum is the optimal threshold value? In summary, it is not possible to find a reliable way for finding the proper threshold value using the histogram. We can conclude that any algorithm that tries to find the threshold value has to find it not only by using the histogram but also by considering the shape of the segmented region after thresholding.

On the other hand, the proposed active contour models and level-set methods work directly with the gradient of the smoothed image. Since their results are very close to each other, we just consider the active contour model. In this model, the contour is specified by a finite set of points *v*_*i*_ = (*x*_*i*_, *y*_*i*_),  *i* = 1 ⋯ *N*. Also, the optimization process is defined through the following energy function:

(20) $$\begin{array}{c} E=\overset{N}{\underset{i=1}{\sum }}E_{\text{external}}\left(v_{i}\right)+E_{\text{internal}}\left(v_{i}\right). \end{array}$$

In (20), *E*_internal_ accounts for the curvature and continuity of the contour, and *E*_external_ represents the energy of the image forces. 

Usually, these terms are defined as follows:

(21) Eexternal(vi)=−|∇Gaussian(σ)∗I(vi)|α,Einternal(vi)=βEcurvature(vi)+γEcontinuity(vi),Ecurvature=||vi−vi−1||2,Econtinuity=||vi+1−2vi+vi−1||2.

In (21), *α*, *β*, and *γ* are user-defined values. Gaussian  (*σ*) is a Gaussian kernel, and ∗ is the convolution operator. The algorithm starts with an initial contour and refines it according to (20) until no change occurs in the contour. There are two important factors for any active contour model. The first factor is the value of *α*, *β*, and *γ*, and the second factor is the initial contour.

**Figure 18:**
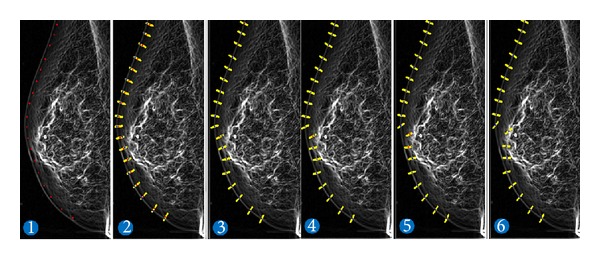
An example of active contour-based segmentation.

Assuming that *α* = 1, *β* = 0, and *γ* = 0, we put an initial contour on a smoothed mammogram and applied the optimization algorithm of the active contour model. This is shown in Figure 18. It is clear from the figure that the initial contour is placed near the actual contour (image 1). After the first iteration (image 2) of the optimization, the contour points *v*_*i*_ (red dots) have moved along their normal towards a point with dominant gradient (blue dots). Since *β* = 0 and *γ* = 0, the algorithm greedily seeks for points with maximum gradients. Finally, the algorithm finishes at the 6th iteration. At this stage, all points of the contour are placed on the points with maximum gradients.

Now, consider that we give nonzero values for parameters *β* and *γ*. This discourages the algorithm from sharp transitions. Also, it tried to keep the distances between consecutive points as equal as possible. By increasing the values of these parameters, the smoothness of the contour is increased. Hence, sharp changes cannot occur in the contour.

Determining the values of these parameters is time consuming, and it can vary for different mammograms. For example, as it is shown in Figure 18, there are tissues with high gradients near the nipple region. The gradient of this area is much higher than the gradient of the actual boundary. Hence, the points of the contour move towards this tissue rather than the boundary. This movement will propagate through *E*_internal_ to other points and will affect their position. As a result, the extracted boundary will be far from the actual boundary.

In addition, the initialization of the contour is a hard problem. In the proposed methods, the authors have initialized the active contour model using a threshold-based method. As we mentioned before, threshold-based methods have their own weaknesses, and they do not have high success rates.

However, as we showed in our experimental results, our algorithm is able to find an accurate boundary using just texture information. By adding the smoothness factor to this algorithm, smoother boundaries are found. Here, instead of using just raw gradient data or pixel intensities, we utilized texture properties of the surrounding region of the pixel.

Also, the only parameter in our algorithm is the size of the texture window. It is not a sensitive parameter like the threshold value or the parameters of active contour models, and it can be approximated easily. Even, if it is considered an important parameter, we could remove its effect by analyzing the mammogram in a scale space. Other parameters of our probability model are obtained through machine-learning techniques.

**Figure 19:**
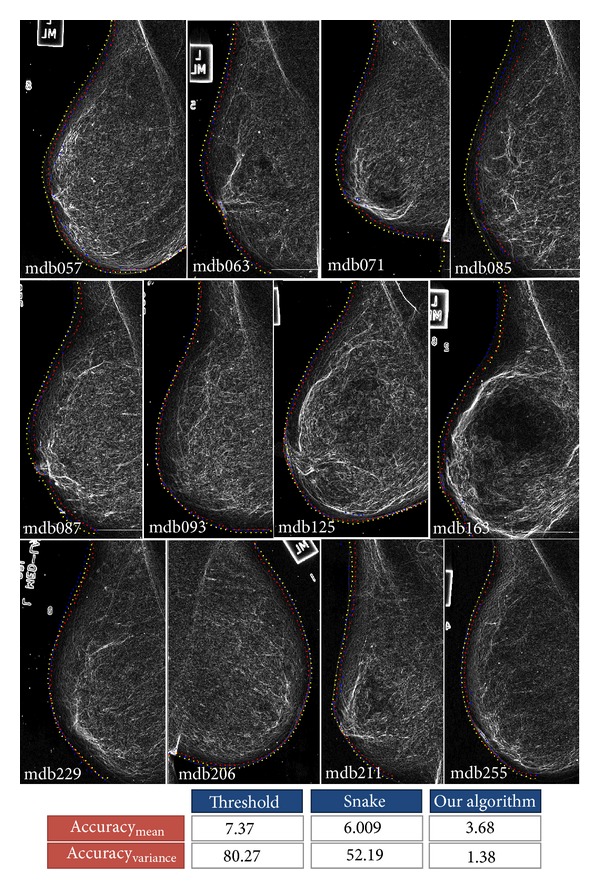
Comparing our algorithm with threshold-based and active contor model methods. The yellow boundary is that found by our algorithm, the blue boundary is that found by the snake method, and the red boundary is the one obtained by the threshold-based method.

With the aim to compare our algorithm with previous methods, we implemented the automatic threshold selection method in [15] which finds these values using shape information of the extracted breast region instead of directly using histogram information. In addition, we used a toolbox which has implemented the three different active contour models. The snake is initialized using threshold-based methods and is refined until it reaches its local minimum. At the same time, we also applied our method on test images (37 images in our case) and compared their results with ground-truth boundaries. The results are shown in Figure 19.

According to this figure, the results of applying the snake model on images mdb093, mdb125, mdb071, mdb063, and mdb206 are close to our algorithm, but the threshold-based method could not find accurate boundaries. Numerical results show 50% and 38% improvement in the results obtained by our method with regard to the snake and threshold-based methods, respectively. However, it should be noted that there are situations in which threshold-based method produces accurate results, but, even in these cases, the results are very close to ours. Another important aspect about the boundary extraction algorithm is its *stability*. In other words, the accuracy of the algorithm must be very close when it is applied on all of the images. To analyze the stability of the algorithms, the variance of acc_mean_ was computed. It is clear from the results that our algorithm is stable in comparison with the two other methods. Statistically, we can see 86% and 83% improvement in the standard deviation of the algorithms.

**Figure 20:**
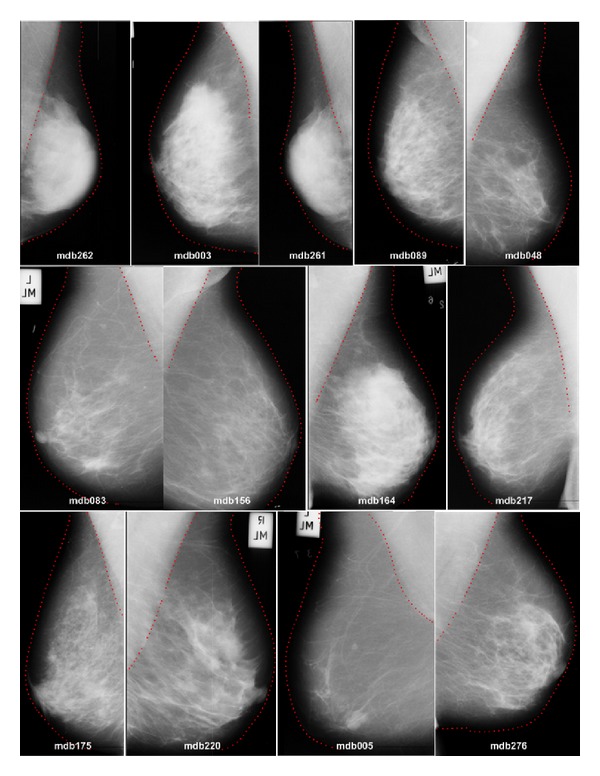
Result of applying the algorithm on several images from mini-MIAS database.

Finally, Figure 20 shows some global results produced by our proposed method. This figure depicts the extracted breast and pectoral muscle boundaries for a variety of mammograms (fatty, fatty glandular, and dense glandular mammograms). From these illustrations, it is feasible to appreciate the accuracy of the algorithm presented in this paper to extract the breast region from the background and the pectoral muscle.

## 5. Conclusion

Extracting the breast boundary in mammograms is a difficult task that has captured the attention of the scientific community. In this paper, we have reviewed the available methods to extract breast boundaries in mammograms, namely, those based on image processing techniques and those based on deformable models, and we have described their pros and cons.

We have proposed a probabilistic approach for solving some of the problems of the current methods. Our approach comprises features of texture, smoothness, and prior probability models. Instead of using raw gray-level values or the gradient of the pixels, we used local binary pattern features to describe the properties of each pixel in the image. The contour is extracted by a region growing strategy, and an initial point in the breast contour is found by classifying the pixels as boundary or nonboundary and selecting the ones with the highest probability of being boundary.

To estimate the probability of a pixel from being part of the boundary, a support vector machine is used. Our basic idea is to use the SVM score as the input for a logistic regression model and, then, find the probability by minimizing a function. Since the core of the algorithm relies on a logistic function, we can expect that there are not sharp transitions for high probabilities.

Experimental results on test data show that our method is able to extract the breast boundary accurately. We have evaluated the importance of the smoothness and prior knowledge components, and we have showed that they are necessary for finding precise boundaries. Also, we have compared our methods against several off-the-shelf approaches, and we have demonstrated the advantages of our method, in terms of both accuracy and stability.
